# Comparison of the Effectiveness of Three Different Acupuncture Methods for TMD-Related Pain: A Randomized Clinical Study

**DOI:** 10.1155/2021/1286570

**Published:** 2021-11-30

**Authors:** Emanuela Serritella, Gabriella Galluccio, Alessandra Impellizzeri, Paola Di Giacomo, Carlo Di Paolo

**Affiliations:** Department of Oral and Maxillofacial Sciences, Sapienza University of Rome, Rome, Italy

## Abstract

**Purpose:**

This study aimed to compare the effectiveness of three acupuncture methods for temporomandibular disorders- (TMDs-) related pain.

**Materials and Methods:**

Different locations of pain, according to DC/TMD clinical assessment, were considered: temporomandibular joint (TMJ), masticatory muscles, head, and neck. Sixty patients were assigned randomly to one of three treatment groups (20 patients in each): group BA received body acupuncture, group EA received electroacupuncture, and group CA received acupuncture + cupping. The groups were compared in terms of pain (verbal numeric scale), pain-related disability (Brief Inventory Pain, BPI), and impression of the treatment's effectiveness (Patients' Global Impression of Improvement Scale, PGI-I). These were recorded before sessions of acupuncture treatment (T0), after 8 sessions of acupuncture treatment (T1), and after 4 weeks of follow-up after treatment (T2). The between-group and within-group differences in the data were analyzed statistically. The baseline characteristics were similar in all groups (*p* > 0.05).

**Results:**

Significant improvements were noted in all types of pain compared to baseline values in all groups (all *p* < 0.05). No significant differences were noted in the improvement of TMDs-related pain according to the different acupuncture techniques (all *p* > 0.05). All acupuncture methods used resulted to be significantly effective in improving the pain-related interference in the patient's common activities and quality of life. EA resulted to be significantly more effective than BA and CA in improving the interference of pain with patients' mood (*p*=0.015) and quality of sleep (*p*=0.014).

**Conclusion:**

BA, EA, and CA are all effective acupuncture methods in reducing pain and pain interference with common activities and quality of life in patients affected by TMD.

## 1. Introduction

Temporomandibular disorders (TMDs) are considered as one of the major causes of orofacial pain [1]. Pain related to TMDs is typically reported in the chewing muscles, preauricular area, or the temporomandibular joint (TMJ) [1, 2]. Often, many patients do not show localized pain but a more complex symptomatology, including headache, cervical pain, atypical facial pain, and head and neck muscle hypersensitivity [3, 4]. The presence of these symptoms may worsen the quality of life of patients and interfere with their emotional and social lives [5]. Due to the wide variety of clinical manifestations of TMDs-related pain, its treatment involves different therapeutic methods, such as splint therapy, medication, surgical therapy, physical therapy, low-level laser therapy (LLLT), transcutaneous electrical nerve stimulation (TENS), ultrasound, vibrational therapy, psychological support, and, increasingly in recent times, acupuncture [6–8].

Although many of acupuncture's physiological and neurological mechanisms are still unknown, the efficacy of acupuncture for pain therapy has been well established [9, 10]. In many clinical studies, acupuncture has been proven to be an effective form of pain management, particularly pain of musculoskeletal origin, including TMDs [9, 11–14]. Acupuncture comprises a wide range of treatment techniques, methods of point stimulation, and devices [15]. *Body acupuncture* is the most common type of acupuncture treatment; it involves the insertion of needles into the selected acupoints, which are manually stimulated by the operator until the achievement of the proper feel of the needling with acupuncture called “Deqi.” This method is actually the most investigated in the orofacial pain field, followed by laser acupuncture, as well as different microsystem acupuncture methods, such as ear, scalp, mouth, and fingers [16–20]. *Electroacupuncture* is a form of acupuncture extensively studied for its analgesic effects [21–23]. This technique involves electrical stimulation of needles, and its growing use in pain management is supported by scientific research demonstrating the differential modulation of endogenous opioids by electrical stimulation of varying frequencies [23]. Few studies investigated the effects of electroacupuncture on TMDs-related pain; although it has been proven to provide significant analgesia, its role in the management of TMDs has not been fully established [22, 23]. *Cupping therapy* belongs to traditional Chinese medicine (TCM), dating back at least 2,000 years. It consists in using one of several kinds of cups (bamboo cups, glasses, or earthen cups) placing them on the desired acupoints or sore spots on patients' skin, producing hyperemia or hemostasis, which results in a therapeutic effect [24]. This method resulted to be effective for treating several pain conditions, especially when combined with other treatments, but its use in the orofacial pain field is poorly documented [24, 25]. A single study by Choi et al. [24] analyzed the effect of acupuncture combined with medicated cupping therapy, reporting this method as effective in treating pain related to TMDs.

Although many studies have assessed the significant effects of different types of acupuncture treatment on TMD-related pain, the conclusions are inconsistent. In particular, due to the lack of direct comparison of different methods of treatment, it is not conducive to the choice of clinical application and the implementation of the best treatment. Therefore, the aim of this clinical study was to evaluate and compare the effectiveness of three different methods of acupuncture treatment (body acupuncture, electroacupuncture, and acupuncture + cupping) in alleviating pain and their interference in common activities and quality of life of patients affected by temporomandibular disorders.

## 2. Materials and Methods

This clinical study was conducted at the Clinical Gnathology Unit of the Department of Oral and Maxillofacial Sciences at the “Sapienza” University of Rome. The study was approved by the Institutional Ethics Committee (N.47/19/0001155); all patients signed an informed consent document before participating in the study.

During the period of February 2019–March 2021, 466 subjects under observation in our department were assessed for eligibility. All patients were screened for temporomandibular disorder (TMD) by specialists in the field and calibrated in using the DC/TMD diagnostic criteria [1]. Criteria for inclusion in the study were as follows: (1) diagnosis of at least one of the following kinds of pain at the craniocervicomandibular level, according to the DC/TMD clinical assessment procedure for the differentiation of pain location in the craniofacial area [1]: TMJ pain, masticatory muscle pain, headache, and neck pain; (2) diagnosis of at least one of the following TMDs (according to Axis I of DC/TMD classification): Myalgia (ICD-9 729.1), Arthralgia (ICD-9 524.62), Headache Attributed to TMD (ICD-9 339.89, ICD-9 784.0), and Disc Displacement with Reduction (ICD-9 524.63); (3) familiar pain intensity greater than or equal to 30 on the verbal numeric scale (VNS); (4) frequency of familiar pain greater than or equal to 1 time/week; and (5) availability to participate in the study. Patients meeting the following exclusion criteria were excluded from the study: (1) diagnosed with widespread pain; (2) chronic use of analgesic medications; (3) diagnosis of Disc Displacement without Reduction Joint disorders (ICD-9 524.63) and/or Degenerative Joint Diseases (ICD‐9 715.18); and (4) receiving ongoing gnathological treatment.

### 2.1. Randomisation and Allocation Concealment

Since there were no data available from other clinical studies about the comparison of different acupuncture methods for the treatment of TMD-related pain, patients were recruited using convenience sampling. The patients who met the inclusion criteria were assigned randomly to three different acupuncture treatment groups. An independent investigator used a random number generator (Research Randomizer©) to allocate participants into each group by block randomisation in a 1 : 1 : 1 ratio. The results of random allocation were sealed, such that they cannot be seen from outside, stored, and managed at the Clinical Gnathology Unit. Before treatment, a random envelope was opened in front of the enrolled participant by the treatment provider. The random allocation numbers assigned to each group were recorded in an electronic chart, and no changes were allowed after allocation.

### 2.2. Blinding

Given the characteristics of the treatments, this study does not allow the participant or treatment provider to be blinded to their group; thus we used a single-blinded design, where only the assessors were blinded. Participants were assessed away from the acupuncture treatment area by investigators who did not participate in treatment procedures and were blinded to the treatment group. The statistician was not involved in randomisation and analyzed the data without having access to information about allocation.

### 2.3. Acupuncture Treatment Groups

The treatment protocol was developed according to the treatment methods used in traditional Chinese medicine (TCM) [14, 15], with the selection of application points based on TCM principles and previous studies [16, 18, 22, 25].

#### 2.3.1. Body Acupuncture (BA Group)

Patients assigned to the first group were treated with body acupuncture (BA). The acupuncture points used were ST6 (Jiache), ST7 (Xiaguan), GB20 (Fengchi), BL10 (Tianzhu), LI4 (Hegu), ST36 (Zusanli), SP6 (Sanyinjiao), and LR3 (Taichong). After asepsis of the skin with 70% alcohol at the needle penetration site, the needles were inserted bilaterally. The needles were disposable and sterilized, individually packed, with size of 0.25 × 25 mm (TEWA, asia-med GmbH, Pullach, Bavaria, Germany). The depth of needle penetration varied considering the anatomical differences of the application sites in each patient. The needle was manipulated clockwise and counterclockwise to achieve the proper feel of needling with acupuncture called “Deqi.”

#### 2.3.2. Electroacupuncture (EA Group)

Patients assigned to the second group were treated with electroacupuncture (EA). The acupuncture points used in this group were the same acupoints selected in the BA Group, as well as the needle penetration procedure and timing, and the needle's size and characteristics. In the EA group, an electrical apparatus (Hwato SDZ-II, Suzhou Medical Appliance Factory, Suzhou, Jiangsu, China) producing a dense-dispersed wave with a frequency of 1/100 Hz was connected to the needles with alligator clips to stimulate pairs of needles inserted at ST36-SP6 and LI4-GB20. The fixed current intensity was uniformly 0.2 mA.

#### 2.3.3. Acupuncture + Cupping (CA Group)

Patients assigned to the third group were treated with acupuncture combined with cupping therapy (CA). The acupuncture points used in this group were the same acupoints selected in BA and EA groups, as well as the needle penetration procedure and timing, and the needle's size and characteristics. In the CA group, at the end of the body acupuncture session, the cupping was carried out with sterile glass cups (Mayfair Medical Supplies Ltd., Kowloon, Hong Kong, China), with size of 3 cm, at the affected side, in correspondence to the acupoints ST6 and ST7. According to the classical method of “retained cupping,” the practitioner used the flaming heating power to achieve suction; the glass cups were retained for about 1-2 minutes and then were detached. The cupping procedure was carried out repeatedly for 10 min.

In all groups (BA, EA, and CA), the needles remained in place for 30 minutes and were then removed. The therapies consist of 8 sessions, administered over 4 weeks, twice a week. The needles were inserted by the same licensed acupuncturist and specialist in orofacial pain with 7-year experience (E. S.) in all treatment groups.

### 2.4. Outcome Measures and Data Analysis

The symptoms evaluated for all patients were the following: temporomandibular joint (TMJ) pain, masticatory muscle pain, headache, and neck pain. Each type of pain was measured at the following times:

- T0: Baseline, before acupuncture treatment
- T1: End of treatment, 4 weeks after T0 (after the last acupuncture session)
- T2: Short-term follow-up, 4 weeks after T1

The 0–100 verbal numeric scale (VNS) was used to measure pain self-assessment, with 0 indicating “no pain” and 100 “the worst imaginable pain.”

At the same times (T0, T1, and T2), all patients completed the following questionnaires, in order to evaluate the general pain-related disability and the impression of the treatment's effectiveness: Brief Inventory Pain (BPI) and Patients' Global Impression of Improvement (PGI-I) Scale [1].

The primary outcome of interest was the pain level and its variation over T0, T1, and T2 in all groups. The secondary outcomes were general pain-related disability (BPI) and the patient's impression of the treatment's effectiveness (PGI-I).

Data analysis was performed with SPSS (version 23) statistical processing software. Descriptive analyses and the Chi-square test were used to compare the patient characteristics. One-way ANOVA on ranks (Kruskal-Wallis test) was used to test differences at the same time interval between groups. Bonferroni-corrected post hoc tests were used for multiple comparisons. Friedman test was used to test changes over three time intervals in the same group. Comparisons of two time intervals were performed with the Wilcoxon signed-rank test. The level of significance was set at *p* < 0.05.

## 3. Results

**Figure 1:**
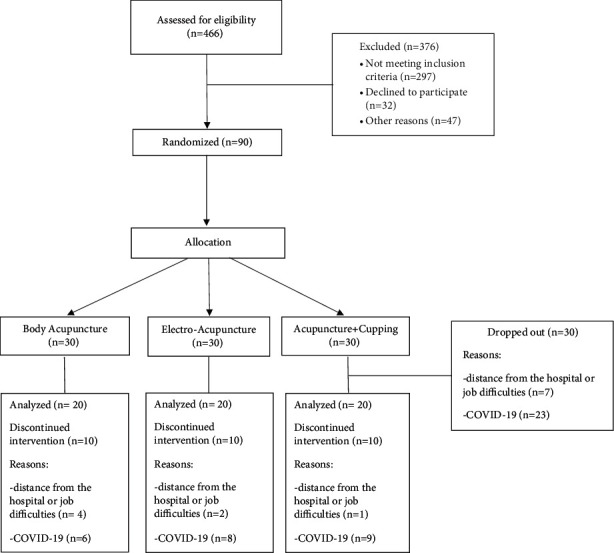
Flow diagram of patient enrollment and interventions.

376 patients were excluded according to the inclusion/exclusion criteria. The resulting study sample consisted of 90 patients, 28 males (31.1%) and 62 females (68.9%), with an average age of 46.93 years. The patient enrollment and intervention process followed the STRICTA (Standards for Reporting Interventions in Clinical Trials of Acupuncture) criteria and is shown in Figure 1.

From the expected sample of 90 suitable patients, 30 were excluded from the analysis for discontinued intervention, mainly due to the impossibility in providing acupuncture treatments caused by COVID-19 pandemic in the period of March–November 2020 (Figure 1).

The resulting study sample therefore consisted of 60 patients (20 patients in each group). The baseline characteristics of the study groups, including age and gender, are shown in Table 1. There were no differences between the groups in terms of baseline characteristics (*p* > 0.05) (Table 1).

No significant adverse effects were seen with respect to the procedure itself. A total of two adverse events were recorded: 12 in the EA group (4 needling pain after treatment and 8 hematomas), 10 in the BA group (3 needling pain after treatment and 7 hematomas), and 11 hematomas in the CA group. These adverse events could remit spontaneously within 1 week. No other side effects or complications were evident and all patients tolerated the treatment well.

### 3.1. Evaluation of Pain Scores

The baseline pain VNS scores in the three groups were comparable for all different pains considered (all *p* > 0.05) (Table 1).

After the treatments (T1) and during the subsequent follow-up visit (T2), no significant differences were observed between the groups (all *p* > 0.05).

**Figure 2:**
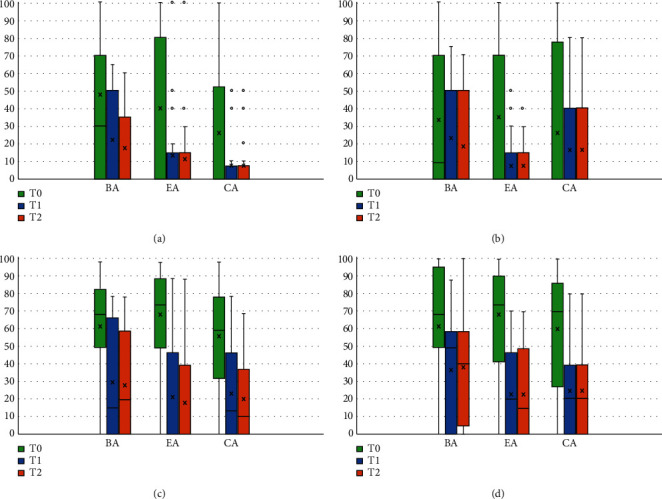
Pain distribution between BA, EA, and CA at T0, T1, AND T2: (a) TMJ pain; (b) muscle pain; (c) headache; (d) neck pain.

Figure 2 shows the quantitative results related to pain (VNS scale), subdivided by the four types of pain analyzed (TMJ pain, masticatory muscle pain, headache, and neck pain). These results show that, in each acupuncture treatment group analyzed (BA, EA, and CA), pain shows average lower values after the treatment (T1) and at the follow-up visits (T2), compared to T0 (Figure 2).

Within-group analyses showed significant improvements in pain VNS values at T1 and T2 compared to baseline values (T0) in all study groups (all *p* < 0.05), except for TMJ pain in EA group and muscle pain in BA group (Table 2).

Wilcoxon signed-rank test for the comparison of baseline (T0) and after treatment (T1) scores and follow-up (T2) scores resulted to be significant in all study groups for all types of pain considered (all *p* < 0.05) and particularly significant for headache (all *p* < 0.001) and neck pain (*p* < 0.001 in EA and CA groups). The results of this analysis and the exact *p* values are shown in Table 2.

### 3.2. Evaluation of Pain-Related Disability (BPI)

Concerning the evaluation of the pain-related interference in the patient's common activities and quality of life after the treatment (T1) and during the subsequent follow-up visit (T2), no significant differences were observed between the groups (all *p* > 0.05), except for the variable mood (*p*=0.015). Interference values were significantly higher in BA and CA groups than in EA group for the variables “mood” and “relations with other people” after the treatment (T1) and at follow-up visit (T2) (Table 3).

Within-group analyses showed significant improvements in interference values at T1 and T2 compared to baseline values (T0) in all study groups (all *p* < 0.05), except for the variable “walking ability” (Table 3).

Wilcoxon signed-rank test for the comparison of baseline (T0) and after treatment (T1) scores and follow-up (T2) scores resulted to be significant in all study groups for all variables considered (all *p* < 0.05), except for the variable “walking ability.” It resulted to be particularly significant for the variables “general activity,” “mood,” and “sleep” (*p* < 0.001) in EA and CA groups. The results of this analysis and the exact *p* values are shown in Table 3.

### 3.3. Treatment Effectiveness (PGI-I)

The results of patients' self-evaluation of treatment effectiveness using the PGI-I Scale are shown in Table 4.

## 4. Discussion

The present study compared the effectiveness of body acupuncture, electroacupuncture, and a combination of body acupuncture with cupping therapy in the treatment of the main types of TMD-related pain: TMJ pain, masticatory muscle pain, headache, and neck pain. Based on the results, all three treatment methods yielded significantly improved outcomes regarding all types of pain considered compared to baseline, reinforcing the evidence that acupuncture is an effective treatment in patients suffering from pain with TMD origin. No significant differences emerged from the between-group analysis, suggesting that there is no specific guidance in selecting one of these three methods based on the TMD-related pain to be treated and thus the referral diagnosis. These results may be explained by the fact that, in the three study groups, different methods of needle stimulation were applied to the same acupuncture points (acupoints) in order to evaluate any differences based only on the type of needle stimulation, not on the treatment principle or scheme. In light of the TCM principle of “treating with the least effort,” using the classical somatic acupuncture stimulation alone can be effective in improving the pain symptoms of the dysfunctional patient. Moreover, body acupuncture does not require any accessory tool/instrument unlike the other two methods used, resulting to be the most easily accessible and the most favorable in terms of cost/benefit. However, despite the lack of evidence of statistical significance, several differences can be highlighted among the results obtained in the three study groups, depending on the type of pain treated.

*Body acupuncture* represents the classic and most common stimulation method of acupuncture and the most frequently mentioned for the treatment of orofacial pain. It involves the insertion of needles into the selected acupoints, which are manually stimulated by the operator. *Zotelli* et al. [17] verified the effectiveness of body acupuncture in the treatment of pain of muscular and mixed origin in patients with TMD, showing an improvement of pain in the treated patients. A review by *Fernandez et al.* [16] evaluated the effectiveness of various body and laser acupuncture treatments for temporomandibular disorder myofascial pain, showing that both of these techniques can be effective in relieving patients' signs and symptoms. These findings are consistent with those of the present study, since the patients belonging to the BA group reported a remarkable decrease of all types of pain analyzed. Although the therapeutic efficacy of body acupuncture is mostly reported on muscle pain, in this study, it was the only type of pain that did not exhibit a statistically significant decrease when comparing the patient's average values both after treatment (T1) and at the subsequent follow-up visit (T2) (*P*_T_ = 0.118). Furthermore, patients of the BA group reported statistically significantly lower average values of TMJ pain (*P*_T_ = 0.004) overall, compared with patients of the CA group (*P*_T_ = 0.047) and especially those of the EA group (*P*_T_ = 0.247). Only one previous study by *Corcos and Brandwein (1976)* focused on the effects of body acupuncture treatment for pain related to the temporomandibular joint in 46 patients affected by rheumatoid arthritis and osteoarthritis. This study also pointed out the value of the body acupuncture method in improving TMJ pain [26]. The other two study groups (CA group and EA group) were characterized by the use of the same therapeutic scheme and procedure as the BA group but adding a “supplemental” stimulation to the selected acupoints. From the results obtained, this addition seems to determine a greater effectiveness in the treatment of pain and especially for headache and neck pain (all *p* < 0.001). Furthermore, both patients belonging to the EA and CA groups reported more significant results compared to patients treated with body acupuncture alone for all types of pain considered, except for TMJ pain.

The addition of *cupping therapy* to body acupuncture was found to be the most effective treatment method in the management of muscle pain. This evidence is consistent with the majority of systematic reviews and RCTs to date which suggest a favorable effect of cupping for pain, especially tension headache and musculoskeletal pain [24]. The negative pressure applied to the skin during the cupping procedure has been proven to induce muscle relaxation and changes in local tissue structures and in blood circulation, significantly reducing peripheral and local P substance and inflammation and thus resulting in pain reduction [24]. *Han* et al. [25] compared the therapeutic effect of medicated cupping and acupuncture combined with medicated cupping in 120 TMD patients, reporting a significant improvement of TMDs' signs and symptoms in both groups after a treatment course of 10 days (*p* < 0.01). In line with the present study, the authors suggested that the combination of the two treatments leads to superior clinical outcomes, compared to the use of single medicated cupping therapy. However, it is difficult to make a comprehensive comparison with the results obtained in this research. The authors did not specify the type of pain treated or the TMD diagnosis of these patients; moreover, the cupping therapy procedure involved the addition of medicinal herbal substances.

To our knowledge, this is the first study to analyze the effects of the classic technique of dry retention cupping combined with acupuncture on the most common types of pain associated with TMD. Given the positive results obtained, the need for further studies becomes evident to deepen and better define the therapeutic potential of this ancient medical practice in relieving pain related to TMD.

*Electroacupuncture* is considered a particularly effective method of acupuncture for the treatment of persistent tissue and nerve injuries, chronic pain, and visceral pain, as addressed by several research studies conducted within the last decade [21, 22, 27]. Zhang et al. [27] suggested that the electroacupuncture mechanism of action in relieving pain is the result of activation or inhibition of various bioactive chemicals in peripheral, spinal, and supraspinal pathways. Despite its popularity in pain management, few studies investigated the clinical effects of electroacupuncture on TMD-related pain. A literature search by *Kuo* et al. [22] yielded to nine publications from Chinese practitioners concerning the use of electroacupuncture for treating TMD symptoms, and all of them reported analgesic efficacy in the treatment of pain, especially of muscular origin. However, the authors highlighted the inconsistency in most of these studies, accentuating the need for more well-designed and long-term studies in this research area. The results obtained in the present study agree with these few lines of evidence, pointing out that this method was particularly effective in reducing pain of muscle origin, as well as headache and neck pain.

While evaluating the effectiveness of treatment methods for TMD, the psychological and emotional status of the patients, as well as their functioning in daily activities, should also be considered. Numerous studies highlighted the association between pain and some social, emotional, and psychological features influencing the quality of life of people affected by TMD [28–30]. Moreover, a particular interest is shown to deepen the correlations between TMD-related pain and quality of sleep and insomnia. When compared to pain-free controls, TMD patients exhibited poorer sleep quality and were mainly categorized as poor sleepers [31, 32]. In addition, poorer sleep quality has been associated with coexisting headaches, body pain, clenching habit, and reduced mouth opening [33]. Connections between mood disorders and TMD were also investigated. Several studies reported high prevalence of symptoms such as anxiety, hostility, anger, paranoid ideation, and especially depression in patients affected by TMD, in particular of muscular origin [34–36]. The presence of depressive symptoms in TMD patients was reported to be related to the presence of a painful condition [36]; the onset or the exacerbation of sufferance and pain in these patients could be generated and perpetuated by the presence of such psychological aspects [34]. In the present study, the impact of pain on all considered activities and quality of life aspects was found to be significantly decreased after the acupuncture treatment and in the short-term follow-up in all three study groups, except for the aspect of “walking ability.” In particular, electroacupuncture was found to be effective in improving the influence of pain on the patient's quality of sleep and mood, with a statistically significant difference compared to body acupuncture and acupuncture combined with cupping. These results are consistent with the evidence defining EA as an effective therapeutic intervention for patients with anxiety, depression, and primary insomnia, capable of improving patients' life and sleep quality without serious adverse effects [37–41]. A multidisciplinary therapeutic approach is needed to address all factors, including sleep and mood alterations, which modulate pain experience. The results of this study indicate that acupuncture, especially electroacupuncture, rehabilitated the patients' ability to perform daily work activities, sleep quality, and emotional and social aspects, rapidly and effectively, up to 1 month after treatment.

Strengths of the present study include the randomized allocation of participants to the treatment groups, the follow-up recording, the differentiation of four types of TMD-related pain recorded, the evaluation of the participants' disability in common activities and quality of life by means of a validated questionnaire, and the evaluation of different methods of stimulation of acupoints using the same therapeutic scheme. Possible weaknesses of the study were the sample size and the duration of the acupuncture treatment. Acupuncture comprises several acupoint stimulation methods, treatment patterns, and timing, allowing the application of individualized therapies. Due to its versatility and special effectiveness in multifactorial diseases, acupuncture is particularly suitable for the treatment of TMD-related pain, which in turn is characterized by a very complex and varied symptomatology. For these reasons, despite the positive results obtained, we think that the patient sample size examined is too limited to present reliable results regarding the comparison of the effectiveness of three different acupuncture methods in reducing TMD-related symptoms. Furthermore, our results correspond to a single course of 4 weeks of therapy. Due to the tendency of TMD to become chronic, a prolonged course evaluation with additional long-term follow-up is necessary.

## 5. Conclusions

- Body acupuncture, electroacupuncture, and acupuncture combined with cupping therapy are all effective methods in reducing pain and pain interference with common activities and quality of life in patients affected by TMD.
- For the first time, the classical method of retained cupping is reported to be effective in the management of TMD-related pain, when combined with body acupuncture.
- Electroacupuncture reduces the interference of pain in patients' mood and sleep quality more effectively than body acupuncture alone or combination with cupping therapy.
- Further studies are needed to confirm the results obtained and to better define the eligibility of one of these methods in improving the complex symptomatology connected with TMD.

## Figures and Tables

**Table 1:** Baseline characteristics of the participants.

Characteristics		Groups			*pvalue*
		BA group (*n* = 20)	EA group (*n* = 20)	CA group (*n* = 20)	
Age, years, mean (SD)		48.25 (15.7)	38.50 (13.67)	48.05 (14.06)	0.0609^*∗*^

Initial pain (VNS), mean (SD)	TMJ	38.75 (38.45)	31.00 (42.66)	24.5 (33.95)	0.5076^*∗*^
	Masticatory muscle	34.00 (37.79)	33.50 (38.45)	39.5 (38.72)	0.8960^*∗*^
	Head	63.25 (31.13)	69.00 (25.73)	56.00 (32.83)	0.3966^*∗*^
	Neck	64.75 (33.54)	64.5 (36.77)	59.25 (33.65)	0.8522^*∗*^

Gender, number (%)	Female	17 (85)	16 (80)	17 (85)	0.8869^*∗∗*^
	Male	3 (15)	4 (20)	3 (15)	

TMD diagnosis, number (%)	Arthralgia	6 (30)	3 (15)	7 (35)	0.4181^*∗∗*^
	Myalgia	13 (65)	13 (65)	8 (40)	
	DDWR^a^	3 (15)	6 (30)	5 (25)	

Side, number (%)	Right	3 (15)	2 (10)	1 (5)	0.5362^*∗∗*^
	Left	3 (15)	1 (5)	1(5)	
	Both	14 (70)	17 (85)	18 (90)	

BA group, body acupuncture treatment; EA group, electroacupuncture treatment; CA group, acupuncture + cupping treatment. ^a^Disc Displacement with Reduction. ^*∗*^*p* value for the comparison of the age and pain distributions among groups (one-way ANOVA). ^*∗∗*^*p* value for the comparison of the gender, diagnosis, and side distributions among groups (Chi-square test).

**Table 2:** Comparisons of pain scores (VNS) within groups after acupuncture treatments (T1) and follow-up visit (T2) (*n* = 20 in each group); mean ± SD values.

Group	T0	T1	T2	*P*_T_	P_0-1_	P_0-2_
*TMJ pain*						
BA group	38.75 ± 38.45	18.00 ± 24.46	16.00 ± 22.57	**0.004**	**0.002**	**0.002**
EA group	31.00 ± 42.66	13.00 ± 26.77	11.00 ± 24.47	0.247	**0.035**	**0.035**
CA group	24.50 ± 33.96	8.00 ± 17.04	6.50 ± 14.24	**0.047**	**0.008**	**0.009**
*Masticatory muscle pain*						
BA group	34.00 ± 37.79	22.50 ± 28.40	21.00 ± 27.51	0.118	**0.022**	**0.014**
EA group	35.50 ± 38.45	9.00 ± 16.83	8.00 ± 14.72	**0.047**	**0.009**	**0.009**
CA group	39.50 ± 38.73	16.50 ± 25.19	16.00 ± 24.58	**0.004**	**0.002**	**0.002**
*Headache*						
BA group	63.25 ± 31.13	30.50 ± 32.84	28.50 ± 31.00	**≤0.001**	**≤0.001**	**≤0.001**
EA group	69.00 ± 25.73	22.50 ± 28.45	17.50 ± 26.73	**≤0.001**	**≤0.001**	**≤0.001**
CA group	56.00 ± 32.83	22.50 ± 25.31	19.00 ± 22.92	**≤0.001**	**≤0.001**	**≤0.001**
*Neck pain*						
BA group	64.75 ± 33.54	37.50 ± 31.44	38.50 ± 32.00	**0.003**	**0.002**	**0.004**
EA group	64.50 ± 36.77	24.00 ± 25.42	23.50 ± 25.19	**≤0.001**	**≤0.001**	**≤0.001**
CA group	59.25 ± 33.65	24.50 ± 25.02	25.00 ± 24.39	**≤0.001**	**≤0.001**	**≤0.001**

VNS, verbal numeric scale; SD, standard deviation. BA group, body acupuncture; EA group, electroacupuncture; CA group, acupuncture + cupping. *P*_T_, *p* value for the within-group comparison (Friedman test); P_0-1_, *p* value for the comparison of baseline (T0) and after 4 weeks (T1) scores (Wilcoxon signed-rank test); P_0-2_, *p* value for the comparison of baseline (T0) and after 8 weeks (T2) scores (Wilcoxon signed-rank test).

**Table 3:** Comparisons of the results of the BPI questionnaire between and within groups *after acupuncture treatments (T1) and at follow-up visit (T2) (n* = *20 in each group)*; mean ± SD values.

Group	T0	T1	T2	*P*_T_	P_0-1_	P_0-2_
*A. General activity*						
BA group	4.45 ± 3.53	2.30 ± 2.66	2.40 ± 2.72	**0.002**	**0.002**	**0.002**
EA group	4.25 ± 2.99	2.00 ± 2.69	1.75 ± 2.40	**≤0.001**	**≤0.001**	**≤0.001**
CA group	4.45 ± 2.11	2.35 ± 2.18	2.45 ± 2.37	**≤0.001**	**≤0.001**	**≤0.001**
*p* ^*∗*^	0.954	0.678	0.529			
P_1-2_	0.785	0.587	0.469			
P_1-3_	0.870	0.782	0.803			
P_2-3_	0.838	0.374	0.249			
*B. Mood*						
BA group	6.25 ± 3.07	3.40 ± 2.94	3.25 ± 2.65	**≤0.001**	**≤0.001**	**≤0.001**
EA group	5.30 ± 2.96	2.05 ± 2.33	1.75 ± 1.94	**≤0.001**	**≤0.001**	**≤0.001**
CA group	6.25 ± 2.29	3.30 ± 2.25	3.35 ± 2.32	**≤0.001**	**≤0.001**	**≤0.001**
*p* ^*∗*^	0.561	**0.015**	0.059			
P_1-2_	0.279	**0.023**	0.074			
P_1-3_	0.623	0.830	0.816			
P_2-3_	0.576	**0.007**	**0.022**			
*C. Walking ability*						
BA group	3.05 ± 3.68	1.40 ± 2.66	1.45 ± 2.78	0.089	0.175	0.196
EA group	1.45 ± 2.50	0.60 ± 1.14	0.60 ± 1.14	0.549	0.491	0.491
CA group	2.30 ± 2.87	1.20 ± 1.85	1.20 ± 1.85	0.091	0.252	0.252
*p* ^*∗*^	0.337	0.675	0.675			
P_1-2_	0.156	0.582	0.582			
P_1-3_	0.671	0.922	0.922			
P_2-3_	0.276	0.340	0.340			
*D. Normal work*						
BA group	4.10 ± 3.51	2.30 ± 3.06	2.35 ± 3.12	**0.011**	**0.003**	**0.004**
EA group	3.60 ± 3.22	1.75 ± 2.47	1.65 ± 2.28	**0.011**	**0.004**	**0.002**
CA group	4.00 ± 2.83	2.00 ± 2.27	2.30 ± 2.41	**≤0.001**	**≤0.001**	**0.003**
*p* ^*∗*^	0.154	0.875	0.582			
P_1-2_	0.097	0.677	0.589			
P_1-3_	0.097	0.988	0.673			
P_2-3_	1	0.641	0.284			
*E. Relations with other people*						
BA group	3.95 ± 3.17	2.15 ± 2.60	2.30 ± 2.77	**0.023**	**0.005**	**0.005**
EA group	2.60 ± 2.76	1.05 ± 1.67	1.10 ± 1.55	**0.048**	**0.007**	**0.008**
CA group	3.70 ± 2.30	2.60 ± 2.54	2.70 ± 2.72	**0.010**	**0.003**	**0.006**
*p* ^*∗*^	0.266	0.092	0.186			
P_1-2_	0.193	0.239	0.390			
P_1-3_	0.753	0.364	0.443			
P_2-3_	0.132	**0.026**	0.053			
*F. Sleep*						
BA group	4.80 ± 3.30	2.95 ± 2.84	3.10 ± 2.83	**≤0.001**	**≤0.001**	**≤0.001**
EA group	4.50 ± 3.47	0.95 ± 1.05	1.75 ± 1.77	**≤0.001**	**≤0.001**	**≤0.001**
CA group	5.05 ± 3.14	3.65 ± 3.12	3.95 ± 3.05	**0.023**	**0.004**	**0.017**
*p* ^*∗*^	0.874	**0.014**	0.062			
P_1-2_	0.775	**0.024**	0.155			
P_1-3_	0.796	0.484	0.317			
P_2-3_	0.614	**0.007**	**0.022**			
*G. Enjoyment of life*						
BA group	5.05 ± 3.89	3.35 ± 3.98	3.35 ± 3.98	**0.047**	**0.015**	**0.015**
EA group	3.05 ± 3.27	1.35 ± 2.50	1.40 ± 2.54	**0.023**	**0.005**	**0.005**
CA group	3.65 ± 3.12	2.30 ± 2.49	2.40 ± 2.68	**≤0.001**	**≤0.001**	**≤0.001**
*p* ^*∗*^	0.222	0.210	0.224			
P_1-2_	0.101	0.142	0.147			
P_1-3_	0.281	0.757	0.790			
P_2-3_	0.419	0.107	0.118			

VNS, verbal numeric scale; SD, standard deviation. BA group, body acupuncture; EA group, electroacupuncture; CA group, acupuncture + cupping. *p*^*∗*^, *p* value for the comparison among groups (one-way ANOVA on ranks); P_1–2_, *p* value for multiple comparisons of BA group and EA group (Bonferroni-corrected post hoc test); P_1-3_, *p* value for multiple comparisons of BA group and CA group (Bonferroni-corrected post hoc test); P_2-3_, *p* value for multiple comparisons of EA group and CA group (Bonferroni-corrected post hoc test). *P*_T_, *p* value for the within-group comparison (Friedman test); P_0-1_, *p* value for the comparison of baseline (T0) and after 4 weeks (T1) scores (Wilcoxon signed-rank test); P_0-2_, *p* value for the comparison of baseline (T0) and after 8 weeks (T2) scores (Wilcoxon signed-rank test).

**Table 4:** Patients' impression of the effectiveness of treatment of the entire study population, according to the PGI-I scale, *after acupuncture treatments (T1) and at follow-up visit (T2) (n* = *60)*; Pt *n* (%).

Patients' impression of the treatment	T1	T2
Very much worse	0 (0%)	0 (0%)
Much worse	0 (0%)	0 (0%)
A little worse	0 (0%)	0 (0%)
No change	9 (15%)	11 (18.3%)
A little better	16 (26.7%)	16 (26.7%)
Much better	17 (28.3%)	16 (26.7%)
Very much better	18 (30%)	17 (28.3%)

## Data Availability

All the data are contained and described within the manuscript. The datasets used and/or analyzed during the current study are available from the corresponding author upon reasonable request.

## Supplementary Materials

Supplementary Materials Table 4: STRICTA 2010 checklist of information to include when reporting interventions in a clinical trial of acupuncture (Expansion of Item 5 from CONSORT 2010 checklist). Table 5: CONSORT 2010 checklist with the Nonpharmacological Trials Extension to CONSORT (with STRICTA 2010 extending CONSORT Item 5 for acupuncture trials).
