# Supplementary material for: Comparison of the Effectiveness of Three Different Acupuncture Methods for TMD-Related Pain: A Randomized Clinical Study
Source: Evid Based Complement Alternat Med. 2021 Nov 30;2021:1286570. doi: 10.1155/2021/1286570 (PMC8632462; doi:10.1155/2021/1286570)
Supplement: Supplementary Materials — Table 4: STRICTA 2010 checklist of information to include when reporting interventions in a clinical trial of acupuncture (Expansion of Item 5 from CONSORT 2010 checklist). Table 5: CONSORT 2010 checklist with the Nonpharmacological Trials Extension to CONSORT (with STRICTA 2010 extending CONSORT Item 5 for acupuncture trials). [file 1286570.f1.doc]

**Table 4: STRICTA 2010 checklist of information to include when reporting interventions in a clinical trial of acupuncture (Expansion of Item 5 from CONSORT 2010 checklist)**

| **Item** | **Detail** | **Reported on page No** |
| --- | --- | --- |
| **1. Acupuncture rationale** | 1a) Style of acupuncture (e.g. Traditional Chinese Medicine, Japanese, Korean, Western medical, Five Element, ear acupuncture, etc) | 5 (Line 125-127) |
|  | 1b) Reasoning for treatment provided, based on historical context, literature sources, and/or consensus methods, with references where appropriate | 5 (Line 125-127) |
|  | 1c) Extent to which treatment was varied | 5-6 |
| **2. Details of needling** | 2a) Number of needle insertions per subject per session (mean and range where relevant) | 5-6 (Line 128-157) |
|  | 2b) Names (or location if no standard name) of points used (uni/bilateral) | 5-6 (Line 128-157) |
|  | 2c) Depth of insertion, based on a specified unit of measurement, or on a particular tissue level | 5-6 (Line 128-157) |
|  | 2d) Response sought (e.g. *de qi* or muscle twitch response) | 5-6 (Line 128-157) |
|  | 2e) Needle stimulation (e.g. manual, electrical) | 5-6 (Line 128-157) |
|  | 2f) Needle retention time | 5-6 (Line 128-157) |
|  | 2g) Needle type (diameter, length, and manufacturer or material) | 5-6 (Line 128-157) |
| **3. Treatment regimen** | 3a) Number of treatment sessions | 6 (Line155-157) |
|  | 3b) Frequency and duration of treatment sessions | 6 (Line155-157) |
| **4. Other components of treatment** | 4a) Details of other interventions administered to the acupuncture group (e.g. moxibustion, cupping, herbs, exercises, lifestyle advice) | 5-6 (Line 128-157) |
|  | 4b) Setting and context of treatment, including instructions to practitioners, and information and explanations to patients | 5-6 (Line 128-157) |
| **5. Practitioner background** | 5) Description of participating acupuncturists (qualification or professional affiliation, years in acupuncture practice, other relevant experience) | 6 (Line 156-158) |
| **6. Control or comparator interventions** | 6a) Rationale for the control or comparator in the context of the research question, with sources that justify this choice |  |
|  | 6b) Precise description of the control or comparator. If sham acupuncture or any other type of acupuncture-like control is used, provide details as for Items 1 to 3 above. |  |

**Table 5: CONSORT 2010 checklist with the Non-pharmacological Trials Extension to CONSORT (with STRICTA 2010 extending CONSORT Item 5 for acupuncture trials)**

| **Section/Topic** | **Item #** | **Checklist item** | **Reported on page No** |
| --- | --- | --- | --- |
| *TITLE AND ABSTRACT* |  |  |  |
|  | 1.a | Identification as a randomized trial in the title | 1  2 |
|  | 1.b | Structured summary of trial design, methods, results, and conclusions. |  |
| *INTRODUCTION* |  |  |  |
| Background and objectives | 2.a | Scientific background and explanation of rationale | 2-4 (Line 49-85)  4 (Line 85-89) |
|  | 2.b | Specific objectives or hypotheses |  |
| METHODS |  |  |  |
| Trial design | 3.a | Description of trial design (e.g., parallel, factorial) including allocation ratio | 4-5 |
|  | 3.b | Important changes to methods after trial commencement (e.g. eligibility criteria), with reasons |  |
| Participants | 4.a | Eligibility criteria for participants | 4 (Line 95-105) |
|  | 4.b | Settings and locations where the data were collected |  |
| Interventions | 5 | The interventions for each group with sufficient details to allow replication, including how and when they were actually administered | 5 (Line 124-158) |
| Outcomes | 6.a | Completely defined pre-specified primary and secondary outcome measures, including how and when they were assessed | 7 (Line 159-172) |
|  | 6.b | Any changes to trial outcomes after the trial commenced with reasons |  |
| Sample size | 7.a | How sample size was determined | 4 (Line 106-109) |
|  | 7.b | When applicable, explanation of any interim analyses and stopping guidelines |  |
| Randomization |  |  |  |
| *Sequence generation* | 8.a | Method used to generate the random allocation sequence | 4 (Line 106-116) |
|  | 8.b | Type of randomization; details of any restriction (e.g., blocking and block size) |  |
| *Allocation concealment* | 9 | Mechanism used to implement the random allocation sequence (e.g., sequentially numbered containers), describing any steps taken to conceal the sequence until interventions were assigned | 4 (Line 106-116) |
| *Implementation* | 10 | Who generated the random allocation sequence, who enrolled participants, and who assigned participants to interventions | 4 (Line 106-116) |
| Blinding | 11.a | If done, who was blinded after assignment to interventions (e.g. participants, care providers, those assessing outcomes) and how | 5 (Line 117-123) |
|  | 11.b | If relevant, description of the similarity of interventions |  |
| Statistical methods | 12.a | Statistical methods used to compare groups for primary and secondary outcomes | 7 (Line173-179) |
|  | 12.b | Methods for additional analyses, such as subgroup analyses and adjusted analyses |  |
| RESULTS |  |  |  |
| Participant flow (A diagram is strongly recommended) | 13.a | For each group, the numbers of participants who were randomly assigned, received intended treatment, and were analyzed for the primary outcome | 8 |
|  | 13.b | For each group, losses and exclusions after randomization, together with reasons |  |
| Implementation of intervention |  |  |  |
| Recruitment | 14.a | Dates defining the periods of recruitment and follow-up | 8 |
|  | 14.b | Why the trial ended or was stopped |  |
| Baseline data | 15 | A table showing baseline demographic and clinical characteristics for each group | 9 (Table 1) |
| Numbers analyzed | 16 | For each group, number of participants (denominator) included in each analysis and whether the analysis was by original assigned groups | 8-9, Table 1 |
| Outcomes and estimation | 17.a | For each primary and secondary outcome, results for each group, and the estimated effect size and its precision (e.g., 95% confidence interval) | 10-16 |
|  | 17.b | For binary outcomes, presentation of both absolute and relative effect sizes is recommended |  |
| Ancillary analyses | 18 | Results of any other analyses performed, including subgroup analyses and adjusted analyses, distinguishing pre-specified from exploratory |  |
| Harms | 19 | All important harms or unintended effects in each group. | 9 (Line 195-199) |
| DISCUSSION |  |  |  |
| Limitations | 20 | Trial limitations, addressing sources of potential bias, imprecision, and, if relevant, multiplicity of analyses | 20 (Line 371-381) |
| Generalizability | 21 | Generalizability (external validity, applicability) of the trial findings | 17 (Line 274-287), 21 (383-393) |
| Interpretation | 22 | Interpretation consistent with results, balancing benefits and harms, and considering other relevant evidence | 17-20 |
| *Other Information* |  |  |  |
| Registration | 23 | Registration number and name of trial registry | 4 (Line 93) |
| Protocol | 24 | Where the full trial protocol can be accessed, if available | 21 |
| Funding | 25 | Sources of funding and other support (e.g., supply of drugs); role of funders | 21 |
